# Honey and Cancer: Sustainable Inverse Relationship Particularly for Developing Nations—A Review

**DOI:** 10.1155/2012/410406

**Published:** 2012-06-17

**Authors:** Nor Hayati Othman

**Affiliations:** Department of Pathology, Universiti Sains Malaysia, Kelantan, 16150 Kubang Kerian, Malaysia

## Abstract

Honey and cancer has a sustainable inverse relationship. Carcinogenesis is a multistep process and has multifactorial causes. Among these are low immune status, chronic infection, chronic inflammation, chronic non healing ulcers, obesity, and so forth. There is now a sizeable evidence that honey is a natural immune booster, natural anti-inflammatory agent, natural antimicrobial agent, natural cancer “vaccine,” and natural promoter for healing chronic ulcers and wounds. Though honey has substances of which the most predominant is a mixture of sugars, which itself is thought to be carcinogenic, it is understandable that its beneficial effect as anticancer agent raises skeptics. The positive scientific evidence for anticancer properties of honey is growing. The mechanism on how honey has anticancer effect is an area of great interest. Among the mechanisms suggested are inhibition of cell proliferation, induction of apoptosis, and cell-cycle arrest. Honey and cancer has sustainable inverse relationship in the setting of developing nations where resources for cancer prevention and treatment are limited.

## 1. Cancer: The Global Epidemic

**Figure 1:**
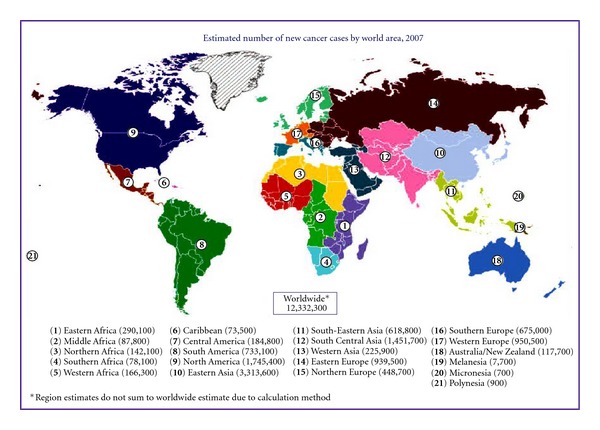
Estimated new cancer cases by world areas (source: Global Cancer Facts and Figures 2007).

Cancer is a global epidemic. In 2008, it was estimated there were 12,332,300 cancer cases of which 5.4 million were in developed countries and 6.7 million were in developing countries [1] (Figure 1). Over half of the incident cases occurred in residents of four WHO regions. The world population increased from 6.1 billion in 2000 to 6.7 billion in 2008 [2]. The increase in populations was much more in developing countries than in developed countries. Even if the age-specific rates of cancer remain constant, developing countries would have a higher cancer burden than developed countries.

Cancer trends are showing upward trends in many developing countries [3–5] and a mixed pattern in developed countries [6–8]. By 2050, the cancer burdencould reach 24 million cases per year worldwide, with 17 million cases occurring in developing countries [9]. Cancers which are associated with diet and life style are seen more in developed countries while cancers which are due to infections are more in developing countries. According to the World Health Organization (WHO), death from cancer is expected to increase to 104% worldwide by 2020. 

While the number of total cancer is increasing, the trend of certain cancers is changing in developed and developing countries. In developed countries, the trend is declining [10] since infections by microorganisms are declining and screening facilities are available. In Singapore, there was an average annual increase of 3.6% for breast cancers in women in the 1988–1992 period [11]. In Qatar, there was a 57.1% rise of cancers 1991–2006 [12], and in Netherlands, there was an increase between 1.9% (females) and 3.4% (males) per year for oesophageal cancer 1989–2003 [13]. 

**Figure 2:**
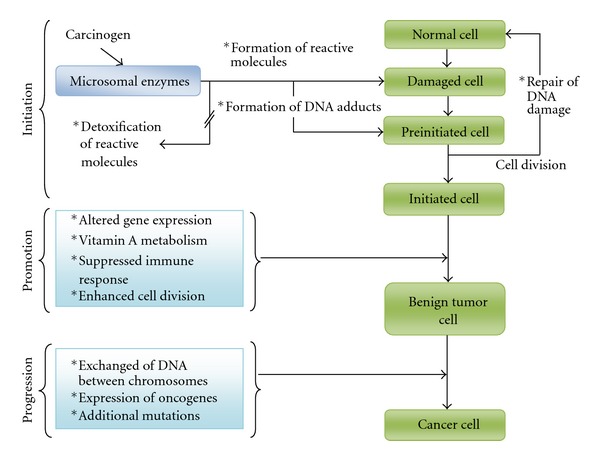
Steps in carcinogenesis. *Steps altered by alcohol consumption (Source: Garro et al. Alcohol Health & Research World 16(1):81–86, 1992).

In order to understand the usefulness of honey in cancer, we need to understand the various factors which could cause cancer. Carcinogenesis is a multi-step process and has multi-factorial causes. Development of cancers takes place long after initiation, promotion, and progression steps (Figure 2) have taken place. The cellular damage could be by one factor or multiplicity of these factors. The latter is more frequent. Cancer development could occur 10–15 years after exposure to the risk factors. 

### 1.1. Life-Style Habits/Diseases as Risks to Cancer Development

**Figure 3:**
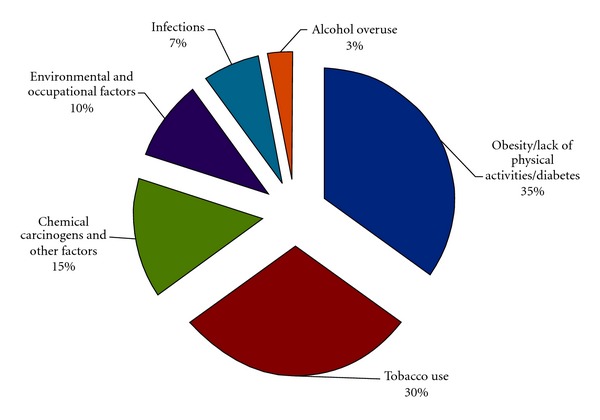
The acquired risk factors of cancer development.

Cancer is caused by genetic damage in the genome of cells. This damage is either inherited or acquired throughout life. The acquired genetic damage is often “self-inflicted” through unhealthy lifestyles. Essentially one-third of cancer is due to tobacco use, one-third due to dietary and lifestyle factors, and one-fifth due to infections. Other factors include chemical carcinogens, environmental pollutants, and alcohol (Figure 3). In the developing countries, cancers caused by infections by microorganisms such as cervical (by human papilloma virus) [14], liver (by hepatitis viruses) [15], nasopharynx (by Epstein-Barr virus) [16], and stomach (by *Helicobacter pylori*) [17] are more common than those in developed countries [18]. While cancers of the prostate, breasts, and colorectal are clearly more prevalent in developed than developing countries, the distinction is not very apparent as that for cancer of the lung which is as prevalent as that in more or less developed nations. Except for breast cancers, the top 5 cancers in males and females of developing nations are due to life-styles or infections [18].

#### 1.1.1. Smoking and Tobacco Use

Association of cancer to cigarette smoking is beyond doubt. The prevalence of smoking is higher in developing than that in developed countries [19]. Smoking is associated with a number of cancers such as larynx, bladder, breasts, oesophagus, and cervix. While in developed countries the prevalence of smoking is decreasing [20], the scenario is the reverse in developing countries. The initiation and the influence to start smoking are similar to those in developed countries [21]. Smoking increases the risk of colorectal carcinomas by 43% [22]. Ever-smokers were associated with an 8.8-fold increased risk of colorectal cancers (95% confidence interval, 1.7–44.9) when fed on well-done red meat diet if they have NAT2 and CYP1A2 rapid phenotypes [23]. No similar association was found in never-smokers [23].

#### 1.1.2. Obesity and Physical Inactivity

Obese subjects have an approximately 1.5–3.5-fold increased risk of developing cancers compared with normal-weight subjects [24]. Obesity is associated in a number of cancers [25, 26] particularly endometrium [27, 28], breasts [29, 30], and colorectal cancers [31]. Adipocytes have the ability to enhance the proliferation of colon cancer cells in vitro [32]. The trend of prevalence of overweight/obesity is rising in many developed and developing countries [33]. In a study conducted in 2005 [34] in the Kota Bharu district in the state of Kelantan Malaysia, the overall prevalence of overweight/obesity was 49.1% [34], much higher than the figure reported earlier in 1996 [35]. In this community, the rise of cancer is exponential in the period from year 2002 to 2007 (143.6% increment) compared to the previous 5-year period of 1996–2001 [36].

Obesity is not a social problem but a disease. The greatest risk is for obese persons who are also diabetic, particular those whose body mass index is above 35 kg/m^2^. The increase in risk is by 93-fold in women and by 42-fold in men [37].

#### 1.1.3. Diabetes Particularly Type 2 as Risk for Cancer Development

Obesity is closely related with diabetes [38]. A community that has high prevalence of obesity also has high prevalence of diabetes [36]. In Kelantan, Malaysia, the prevalence of diabetes in 1999 was 10.5%, and impaired glucose tolerance was 16.5% [39]. Kelantan is ranked highest in prevalence of diabetes in Malaysia in which the overall national prevalence is 8.3% [40], thus it was not a surprise to see a rapid rise of cancer prevalence in the state [36]. According to a review on diabetes, the WHO has estimated that, by 2030, there would be 2.48 million diabetics in Malaysia, a jump of 164% from 0.94 million in 2002 [41]. One of the most common cancers noted in community that has high diabetics and obesity is colorectal cancer [42–45].

In a study of 138 colorectal cancers (CRC) seen in Hospital Universiti Sains Malaysia, 47.8% had metabolic diseases, of which 13.8% were diabetes type 2 [42]. Those diabetics with CRC often have distal cancers [42]. 

### 1.2. Chronic Infections as Risk for Cancer Development

There are a number of microorganisms which could cause cancer. Common viruses causing cancers [46] are Epstein-Barr virus (EBV) [47] (nasopharyngeal carcinomas), human papilloma virus (cervical cancers and other squamous cancers) and Hepatitis B viruses (liver cancers). Viruses are oncogenic after long period of latency [48].

 Bacteria which has been studied to have associations with cancer are *Helicobacter pylori* infections (stomach cancer) [17], *Ureaplasma urealyticum* (prostate cancer) [49], and chronic typhoid carrier (gall bladder cancer) [50]. Chronic fungi infections have also been studied to be associated with cancer [51]. Parasites such as *Schistosoma haematobium* are associated with carcinoma of the urinary bladder; liver flukes *Opisthorchis viverrini* and *Clonorchis sinensis* associated with cholangiocarcinoma and hepatocellular carcinoma. There are three main mechanisms by which infections can cause cancer. They appear to involve initiation as well as promotion of carcinogenesis [52]. Persistent infection within host induces chronic inflammation accompanied by formation of reactive oxygen and nitrogen species (ROS and RNOS) [52]. ROS and RNOS have the potential to damage DNA, proteins, and cell membranes. Chronic inflammation often results in repeated cycles of cell damage leading to abnormal cell proliferation [53]. DNA damage promotes the growth of malignant cells. Secondly, infectious agents may directly transform cells, by inserting active oncogenes into the host genome, inhibiting tumour suppressors or stimulating mitosis [52]. Thirdly, infectious agents, such as human immunodeficiency virus (HIV), may induce immunosuppression [52].

## 2. Low Immune Status as Risk of Cancer Development

### 2.1. Cancer and Aging

The most important change that would occur in the world population in the next 50 years is the change in the proportion of elderly people (more than 65 years): 7% in 2000 to 16% in 2050 [54]. Many cancers are associated with aging. Although age per se is not an important determinant of cancer risk, it implies prolonged exposure to carcinogen [55]. By the year 2050, 27 million people are projected to have cancer. More than half of the estimated number will be residents of developing countries [54]. Aging is also associated with reduced immune system.

### 2.2. Low Immune Status due to Chronic Diseases

Patients who have low immune system are at risk for cancer development. This explains why diabetics are more at risk than non-diabetics to get epithelial cancers. HIV patients are at risk to develop epithelial and nonepithelial cancers. These persons are also at risk to develop multiple chronic infections implying the multiplicity in cancer genesis. Patients with autoimmune diseases are also at risk to develop cancers such as colorectal carcinomas in ulcerative colitis and Crohn's disease and thyroid cancer in autoimmune thyroiditis. 

### 2.3. Chronic Ulcers and Wounds

Chronic ulcers have risk to develop cancer. The most common is Marjolin's ulcer [56], and they are common in developing nations especially in rural areas with poor living conditions [57]. This risk factor is related to chronic infections as most if not all chronic ulcers are not healing because of persistent infections.

## 3. What Is Honey and Why Is It Useful against Cancer? (See Figure 4)

Honey is known for centuries for its medicinal and health-promoting properties. It contains various kinds of phytochemicals with high phenolic and flavonoid content which contribute to its high antioxidant activity [58–60]. Agent that has strong antioxidant property may have the potential to prevent the development of cancer as free radicals and oxidative stress play a significant role in inducing the formation of cancers [61]. Phytochemicals available in honey could be narrowed down into phenolic acids and polyphenols. Variants of polyphenols in honey were reported to have antiproliferative property against several types of cancer [62]. 

## 4. Honey As a Natural Immune Booster

Honey stimulates inflammatory cytokine production from monocytes [63]. Manuka, pasture, and jelly bush honey were found to significantly increase TNF-*α*, IL-1*β*, and IL-6 release from MM6 cells (and human monocytes) when compared with untreated and artificial honey-treated cells (*P* < 0.001) [63]. A 5.8 kDa component of manuka honey was found to stimulate cytokine production from immune cells via TLR4 [64]. Honey stimulates antibody production during primary and secondary immune responses against thymus-dependent and thymus-independent antigens in mice injected with sheep red blood cells and *E. coli* antigen [65]. Consumption of 80 g daily of natural honey for 21 days showed that prostaglandin levels compared with normal subjects were elevated in patient with AIDS [66]. Natural honey has been shown to decrease prostaglandin level, elevated NO production in patients with a long history of AIDS [66]. It was reported that oral intake of honey augments antibody productions in primary and secondary immune responses against thymus-dependent and thymus-independent antigens [67].

These studies suggest that daily consumption of honey improves one's immune system.

## 5. Honey As Natural Anti-Inflammatory Agent

In routine everyday life, our cells may be injured by irritants from outside or within our bodies (by microbes or nonmicrobes). Cellular/molecular injuries result in inflammatory response, the body defense mechanisms in trying to rid of the irritants. In general inflammatory responses are beneficial and protective to us, but at times, inflammatory responses are detrimental to health. Honey is a potent anti-inflammatory agent. Infants suffering from diaper dermatitis improved significantly after topical application of a mixture containing honey, olive oil, and beeswax after 7 days [68]. Honey provides significant symptom relief of cough in children with an upper respiratory tract infection (URTI) [69]. It has been shown to be effective in management of dermatitis and *Psoriasis vulgaris* [70]. Eight out of 10 patients with dermatitis and five of eight patients with psoriasis showed significant improvement after 2 weeks on honey-based ointment [70]. Honey at dilutions of up to 1 : 8 reduced bacterial adherence from 25.6  ±  6.5 (control) to 6.7  ±  3.3 bacteria per epithelial cell (*P* < 0.001) in vitro [71]. Volunteers who chewed “honey leather” showed that there were statistically highly significant reductions in mean plaque scores (0.99 reduced to 0.65; *P* = 0.001) in the manuka honey group compared to the control group suggesting a potential therapeutic role for honey for gingivitis, periodontal disease [72], mouth ulcers, and other problems of oral health [73].

A case report of a patient who had chronic dystrophic epidermolysis bullosa (EB) for 20 years healed with honey impregnated dressing in 15 weeks [74] after conventional dressings and creams failed. This illustrates the usefulness of honey as an anti-inflammatory agent. Chronic inflammatory process has risk of cancer development.

## 6. Honey As Natural Antimicrobials

Everyday we are exposed to all kinds of microbial insults from bacteria, viruses, parasites, and fungi. Honey is a potent natural antimicrobial. The most common infections humans get are from staphylococcal infection. Antibacterial effect of honey is extensively studied. The bactericidal mechanism is through disturbance in cell division machinery [75]. The minimum inhibitory concentration (MIC) for *Staphylococcus aureus* by *A. mellifera* honey ranged from 126.23 to 185.70 mgml^−1^ [76]. Honey is also effective against coagulase-negative staphylococci [77]. Local application of raw honey on infected wounds reduced signs of acute inflammation [78], thus alleviating symptoms. Antimicrobial activity of honey is stronger in acidic media than in neutral or alkaline media [78]. The potency of honey is comparable to some local antibiotics. Honey application into infective conjunctivitis reduced redness, swelling, pus discharge, and time for eradication of bacterial infections [78]. When honey is used together with antibiotics, gentamycin, it enhances anti-*Staphylococcus aureus* activity, by 22% [79]. When honey is added to bacterial culture medium, the appearance of microbial growth on the culture plates is delayed [80]. Mycobacteria did not grow in culture media containing 10% and 20% honey while it grew in culture media containing 5%, 2.5%, and 1% honey, suggesting that honey could be an ideal antimycobacterial agent [81] at certain concentrations.

Honey is also effective in killing hardy bacteria such as *Pseudomonas aeruginosa* (PA) and could lead to a new approach in treating refractory chronic rhinosinusitis [82]. Daily consumption of honey reduces risk of chronic infections by microorganisms. Chronic infections have risk for cancer development.

There are three main mechanisms by which infections can cause cancer. They appear to involve initiation as well as promotion of carcinogenesis [52]. Persistent infection within host induces chronic inflammation accompanied by formation of reactive oxygen and nitrogen species (ROS and RNOS) [52]. ROS and RNOS have the potential to damage DNA, proteins, and cell membranes. Chronic inflammation often results in repeated cycles of cell damage leading to abnormal cell proliferation [53]. DNA damage promotes the growth of malignant cells. Secondly, infectious agents may directly transform cells, by inserting active oncogenes into the host genome, inhibiting tumour suppressors [52]. Thirdly, infectious agents, such as human immunodeficiency virus (HIV), may induce immunosuppression [52].

The effectiveness of honey is best when used at room temperature. Heating honey to 80 degrees for 1 hour decreased antimicrobial activity of both new and stored honey. Storage of honey for 5 years decreased its antimicrobial activity, while ultraviolet light exposure increased its activity against some of microorganisms [78].

Honey also has been shown to have antiviral properties. In a comparative study topical application of honey was found to be better than acyclovir treatment on patients with recurrent herpetic lesions [83]. Two cases of labial herpes and one case of genital herpes remitted completely with the use of honey while none with acyclovir treatment [83]. 

## 7. Honey As Possible Agent for Controlling Obesity

Obese individuals are at risk to develop cancer. There is a close link among obesity, a state of chronic low-level inflammation, and oxidative stress [84]. Obese subjects have an approximately 1.5–3.5-fold increased risk of developing cancers compared with normal-weight subjects [24–26] particularly endometrium [27, 28], breasts [29, 30], and colorectal cancers [31]. Adipocytes have the ability to enhance the proliferation of colon cancer cells in vitro [32]. The greatest risk is for obese persons who are also diabetic, particularly those whose body mass index is above 35 kg/m^2^. The increase in risk is by 93-fold in women and by 42-fold in men [37]. One of the most common cancers noted in community that has high diabetics and obesity is colorectal cancer [42–45].

 In a clinical study on 55 overweight or obese patients, the control group (17 subjects) received 70 g of sucrose daily for a maximum of 30 days and patients in the experimental group (38 subjects) received 70 g of natural honey for the same period. Results showed that honey caused a mild reduction in body weight (1.3%) and body fat (1.1%) [85]. Beneficial effect of honey on obesity is not well established thus far.

## 8. Honey as “Fixer” for Chronic Ulcers and Wounds

Increasing numbers of antibiotic-resistant bacteria has made simple wounds become chronic and non-healing and as such honey provides alternative treatment options [86]. Honey absorbs exudates released in wounds and devitalized tissue [87]. Honey is effective in recalcitrant surgical wounds [88]. It increases the rate of healing by stimulation of angiogenesis, granulation, and epithelialization, making skin grafting unnecessary and giving excellent cosmetic results [89]. In a randomized control trial, Manuka honey improved wound healing in patients with sloughy venous leg ulcers [90]. Honey was shown to eradicate MRSA (Methylene resistant *Staphylococcus aureus*) infection in 70% of chronic venous ulcers [91]. Honey is acidic and chronic non healing wounds have an elevated alkaline environment. Manuka honey dressings is associated with a statistically significant decrease in wound pH [92]. Available evidence in meta-analysis studies indicates markedly greater efficacy of honey compared with alternative dressings for superficial or partial thickness burns [93]. Honey is an inexpensive moist dressing with antibacterial and tissue-healing properties suitable for diabetic foot [94]. The average cost of treatment per patient using honey dressing is much cheaper with conventional dressing [95].

## 9. Honey As Natural Cancer “Vaccine”

Synthetic vaccines like BCG or polio vaccine work by preventing vaccinated subjects from contracting tuberculosis and poliomyelitis. Honey has the element of a “natural cancer vaccine” as it can reduce chronic inflammatory processes, improve immune status, reduce infections by hardy organisms and so forth. Some simple and polyphenols found in honey, namely, caffeic acid (CA), caffeic acid phenyl esters (CAPE), chrysin (CR), galangin (GA), quercetin (QU), kaempferol (KP), acacetin (AC), pinocembrin (PC), pinobanksin (PB), and apigenin (AP), have evolved as promising pharmacological agents in prevention and treatment of cancer [62]. The antioxidant activity of *Trigona carbonaria* honey from Australia is high at 233.96±50.95 microM Trolox equivalents [96]. The antioxidant activity of four honey samples from different floral sources showed high antioxidant properties tested by different essay methods [97]. Dark honey had higher phenolic compounds and antioxidant activity than clear honey [98]. The amino acid composition of honey is an indicator of the toxic radical scavenging capacity [99].

## 10. Honey as Potential Use in “Cancer Therapy”

Honey may provide the basis for the development of novel therapeutics for patients with cancer and cancer-related tumors. Jungle honey fragments were shown to have chemotactic induction for neutrophils and reactive oxygen species (ROS), proving its antitumor activity [67]. Recent studies on human breast [100], cervical [100], oral [101], and osteosarcoma [101] cancer cell lines using Malaysian jungle honey showed significant anticancer activity. Honey has been shown to have antineoplastic activity in an experimental bladder model in vivo and in vitro [102]. 

Honey is rich in flavonoids [62, 103]. Flavanoids have created a lot of interests among researchers because of its anticancer properties. The mechanisms suggested are rather diverse such as various signaling pathways [104], including stimulation of TNF-alpha (tumor necrosis factor-alpha) release [105], inhibition of cell proliferation, induction of apoptosis [106], and cell cycle arrest [107] as well as inhibition of lipoprotein oxidation [108]. Honey is thought to mediate these beneficial effects due to its major components such as chrysin [104] and other flavonoids [109]. These differences are explainable as honeys are of various floral sources, and each floral source may exhibit different active compounds. Though honey has other substances of which the most predominant are a mixture of sugars (fructose, glucose, maltose, and sucrose) [110] which itself is carcinogenic [111], it is understandable that its beneficial effect on cancer raises skeptics. The mechanism on how honey has anti-cancer effect is an area of great interest recently. The effects of honeys on hormone-dependent cancers such as breast, endometrial, and prostate cancer and tumors remain largely unknown. There is a lot we can learn from nature [112]. For example, phytochemicals, such as genistein, lycopene, curcumin, epigallocatechin-gallate, and resveratrol have been studied to be used for treatment of prostate cancer [113]. Phytoestrogens constitute a group of plant-derived isoflavones and flavonoids, and honey belongs to plant phytoestrogen [112, 114]. 

## 11. Conclusion

There is now a sizeable evidence that honey is a natural immune booster, natural anti-inflammatory agent, natural antimicrobial agent, natural cancer “vaccine,” and natural promoter for healing chronic ulcers and wounds; some of the risk factors for cancer development. Bee farming is a lucrative business. Honey and cancer have sustainable inverse relationship in the setting of developing nations where resources for cancer prevention and treatment are limited.

## Figures and Tables

**Figure 4:**
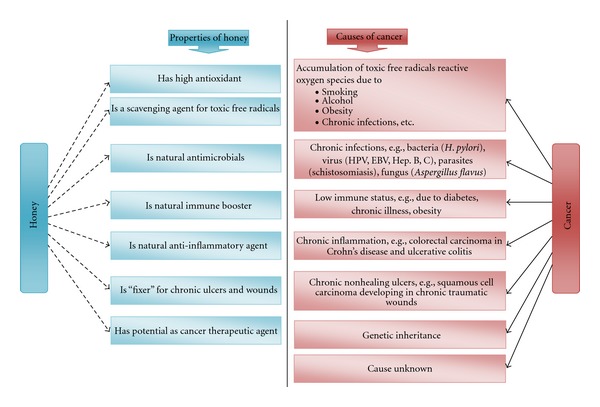
The inverse relationship of honey and cancer.
